# Supplementary material for: Association of Low Alanine Aminotransferase Values with Extubation Failure in Adult Critically Ill Patients: A Retrospective Cohort Study
Source: J Clin Med. 2021 Jul 25;10(15):3282. doi: 10.3390/jcm10153282 (PMC8348471; doi:10.3390/jcm10153282)
Supplement: Supplementary file 1 [file jcm-10-03282-s001.zip › jcm-1267551-supplementary.pdf]

**Supplementary Table S1.** Results of the bivariate analyses of the correlation between short-term (48 hours) extubation outcome and demographic factors, co-morbidities, vital signs, and laboratory test results of 329 patients included in the study.

| | Short-term (48 hours) extubation outcome | | | |
|---|---|---|---|---|
| | **Failure (n=66)** | **Success (n=263)** | **OR (95% CI)** | ***p*-value** |
| **Age, years (IQR)** | 63.6 (54.3-75.4) | 62.3 (46.3-71) | 1.01 (0.99-1.03) | 0.28 |
| **Male gender, n (%)** | 34 (51.5%) | 171 (65%) | 0.57 (0.33-0.99) | ***0.04*** |
| **Body mass index, kg/m$^2$ (IQR)** | 27.3 (24.2-29.3) | 26.7 (23.4-31.2) | 0.98 (0.94-1.04) | 0.55 |
| **Comorbidities** | | | | |
| Diabetes Mellitus, n (%) | 26 (39.4%) | 87 (33.1%) | 1.54 (0.77-3.1) | 0.22 |
| Chronic Kidney Disease, n (%) | 10 (15.2%) | 44 (16.7%) | 0.7 (0.31-1.63) | 0.41 |
| Congestive Heart Failure, n (%) | 20 (30.3%) | 69 (26.2%) | 1.44 (0.68-3.06) | 0.34 |
| Cerebrovascular disease, n (%) | 12 (18.2%) | 40 (15.2%) | 1.3 (0.62-2.72) | 0.48 |
| COPD, n (%) | 10 (15.2%) | 65 (24.7%) | 0.47 (0.22-1.01) | 0.05 |
| Hypertension, n (%) | 34 (51.5%) | 137 (52.1%) | 0.77 (0.39-1.55) | 0.47 |
| Ischemic Heart Disease, n (%) | 16 (24.2%) | 58 (22.1%) | 1.01 (0.47-2.19) | 0.98 |
| **Admission APACHE-II score (SD)*** | 28 (23-33) | 28 (23-32) | 1.02 (0.97-1.07) | 0.39 |
| **Medications** | | | | |

| | | | | | |
|---|---|---|---|---|---|
| | Corticosteroids (during 7 days before extubation), n (%) | 26 (39.4%) | 87 (33.1%) | 1.31 (0.75-2.29) | 0.34 |
| | Muscle relaxants (during index admission), n (%) | 12 (18.2%) | 48 (18.3%) | 1 (0.49-2) | 0.99 |
| | Vasopressors (during 24 hours before extubation), n (%) | 10 (15.2%) | 49 (18.6%) | 0.78 (0.37-1.64) | 0.51 |
| **Length of ICU stay before first extubation trial, days (IQR)** | | 4.68 (2.23-7.35) | 3.53 (1.5-7.54) | 0.99 (0.94-1.05) | 0.45 |
| **Duration of mechanical ventilation, days (IQR)** | | 3.7 (1.73-6.35) | 2.78 (1.08-5.7) | 1 (0.94-1.08) | 0.83 |
| **High-risk of extubation failure, n (%)** | | 42 (63.6%) | 168 (63.9%) | 0.88 (0.41-1.9) | 0.74 |
| **Very high-risk of extubation failure, n (%)** | | 30 (45.5%) | 114 (43.3%) | 1.18 (0.56-2.49) | 0.66 |
| **Laboratory values (last value before extubation)** | | | | | |
| | Albumin, g/dL (IQR) | 2.65 (2.3-3) | 2.9 (2.5-3.2) | 0.61 (0.41-0.9) | ***0.01*** |
| | Creatinine, mg/dL (IQR) | 0.86 (0.6-1.47) | 0.77 (0.63-1.24) | 1.19 (0.92-1.53) | 0.18 |
| | Blood urea nitrogen, mg/dL (IQR) | 25 (14-43.2) | 19.7 (12.7-32) | 1.01 (0.99-1.02) | 0.17 |
| | Hemoglobin, g/dL (IQR) | 9.65 (8.5-11.9) | 10.3 (8.8-11.9) | 0.96 (0.84-1.09) | 0.51 |
| | Sodium, mEq/L (IQR) | 140 (137-145) | 140 (138-143) | 1 (0.96-1.06) | 0.86 |
| | Potassium, mmol/L (IQR) | 3.7 (3.4-4) | 3.8 (3.5-4.2) | 0.45 (0.26-0.78) | ***0.005*** |
| | Phosphorus, mg/dl (IQR) | 3.07 (2.4-3.9) | 3.35 (2.52-4.11) | 0.89 (0.73-1.1) | 0.28 |
| | Magnesium, mg/dl (IQR) | 1.93 (1.71-2.14) | 1.99 (1.77-2.24) | 0.56 (0.25-1.24) | 0.15 |

| | | | | | |
|---|---|---|---|---|---|
| | Brain natriuretic peptide, pg/mL (IQR)** | 1868 (301-3347) | 1230 (432-3875) | 1 (0.99-1) | 0.5 |
| | pH | 7.41 (7.35-7.46) | 7.41 (7.36-7.47) | 0.6 (0.01-25.9) | 0.79 |
| | $pO_2$, mmHg (IQR) | 93 (78-123) | 106 (84-138) | 0.99 (0.99-1) | 0.13 |
| | $pCO_2$, mmHg (IQR) | 48 (42-56) | 45 (39-53) | 1.02 (1-1.04) | 0.13 |
| | Bicarbonate, mmol/L (IQR) | 30.6 (25.8-36.4) | 29.1 (24.8-34.8) | 1.02 (0.99-1.06) | 0.23 |
| | Lactate, mmol/L (IQR) | 1.2 (0.95-1.65) | 1.1 (0.85-1.7) | 0.92 (0.75-1.12) | 0.39 |
| | Ionized calcium, mmol/L (IQR) | 1.1 (1.04-1.16) | 1.09 (1.04-1.14) | 6.47(0.25-167.03) | 0.26 |
| | Hyperglycemia (>180mg/dL) during 48h preceding extubation, n (%) | 37 (56.1%) | 161 (61.2%) | 0.81 (0.47-1.4) | 0.44 |
| **Vital signs (last value before extubation)** | | | | | |
| | Heart rate, bpm (IQR) | 91 (80-110) | 92 (81-107) | 1 (0.99-1.01) | 0.99 |
| | Systolic blood pressure, mmHg (IQR) | 141 (123-156) | 139 (121-158) | 1 (0.99-1.01) | 0.85 |
| | Diastolic blood pressure, mmHg (IQR) | 67 (57-75) | 66 (57-75) | 1 (0.98-1.02) | 0.99 |
| | Core Temperature, °C (IQR) | 36.7 (36.2-37.4) | 36.8 (36.3-37.4) | 0.94 (0.67-1.31) | 0.72 |
| | Oxygen saturation, % (IQR) | 98 (95-100) | 98 (96-100) | 0.96 (0.86-1.09) | 0.56 |
| | Fraction of inspired oxygen, % (IQR) | 40 (35-40) | 40 (35-40) | 1.01 (0.95-1.07) | 0.84 |
| **Muscle related laboratory values (lowest value over the preceding 10 days)** | | | | | |

| | | | | | |
|---|---|---|---|---|---|
| | Alanine transaminase, IU/L (IQR) | 14 (9-21) | 19 (12-26) | 0.96 (0.93-0.99) | ***0.004*** |
| | Creatine phosphokinase, U/L (IQR)*** | 105 (38-234) | 136 (52-475) | 1 (1-1) | 0.2 |

Statistically significant values (*p*< 0.05) are italicized and bolded.

* APACHE-II score was recorded in 239 cases (72.6%)

** Brain natriuretic peptide values were available for 72 patients (21.9%)

*** Creatine phosphokinase values were available for 163 patients (49.5%)

OR- odds ratio; 95% CI- 95% confidence interval; IQR- interquartile range; COPD- chronic obstructive lung disease; APACHE-II score- Acute Physiology and Chronic Health Evaluation II

**Supplementary Table S2.** Results of the bivariate analyses of the correlation between long-term (7 days) extubation outcome and demographic factors, co-morbidities, vital signs, and laboratory test results of 329 patients included in the study.

| | | **Long-term (7 days) extubation outcome** | | | |
|---|---|---|---|---|---|
| | | **Failure (n=83)** | **Success (n=246)** | **OR (95% CI)** | ***p*-value** |
| **Age, years (IQR)** | | 65.7 (55-76.2) | 62.1 (45.7-70) | 1 (1-1.04) | ***0.013*** |
| **Male gender, n (%)** | | 45 (54.2%) | 160 (65%) | 0.64 (0.38-1.05) | 0.08 |
| **Body mass index, kg/m$^2$ (IQR)** | | 27.5 (24.2-30.9) | 26.2 (23.4-31.03) | 1.01 (0.97-1.05) | 0.73 |
| **Comorbidities** | | | | | |
| | Diabetes Mellitus, n (%) | 35 (42.2%) | 78 (31.7%) | 1.57 (0.94-2.62) | ***0.09*** |

| | | | | | |
|---|---|---|---|---|---|
| | Chronic Kidney Disease, n (%) | 14 (16.9%) | 40 (16.3%) | 1.04 (0.54-2.04) | 0.89 |
| | Congestive Heart Failure, n (%) | 26 (31.3%) | 63 (25.6%) | 1.33 (0.77-2.29) | 0.32 |
| | Cerebrovascular disease, n (%) | 15 (18.1%) | 37 (15 %) | 1.25 (0.64-2.41) | 0.52 |
| | COPD, n (%) | 14 (16.9%) | 61 (24.1%) | 0.62 (0.32-1.17) | 0.13 |
| | Hypertension, n (%) | 48 (57.3%) | 123 (50%) | 1.37 (0.83-2.27) | 0.22 |
| | Ischemic Heart Disease, n (%) | 26 (31.3%) | 48 (19.5%) | 1.88 (1.07-3.3) | ***0.03*** |
| **Admission APACHE-II score (SD)*** | | 29.29 (6.66) | 27.24 (6.31) | 1.05 (1-1.1) | ***0.03*** |
| **Medications** | | | | | |
| | Corticosteroids (during 7 days before extubation), n (%) | 32 (38.6%) | 81 (32.9%) | 1.28 (0.76-2.14) | 0.35 |
| | Muscle relaxants (during index admission), n (%) | 17 (20.5%) | 43 (17.5%) | 1.21 (0.65-2.28) | 0.54 |
| | Vasopressors (during 24 hours before extubation), n (%) | 13 (15.7%) | 46 (18.7%) | 0.81 (0.41-1.58) | 0.53 |
| **Length of ICU stay before first extubation trial, days (IQR)** | | 4.87 (2.63-7.72) | 3.34 (1.46-6.77) | 0.99 (0.99-1.01) | 0.49 |
| **Duration of mechanical ventilation, days (IQR)** | | 3.71 (1.74-6.27) | 2.63 (1.01-5.66) | 1.01 (0.95-1.08) | ***0.018*** |
| **High-risk of extubation failure, n (%)** | | 58 (69.9%) | 152 (61.8%) | 1.43 (0.84-1.75) | 0.18 |
| **Very high-risk of extubation failure, n (%)** | | 41 (49.4%) | 103 (41.9%) | 1.36 (0.82-2.23) | 0.23 |
| **Laboratory values (last value before extubation)** | | | | | |
| | Albumin, g/dL (IQR) | 2.7 | 2. 9 | 0.62 | ***0.015*** |

| | | | | | |
|---|---|---|---|---|---|
| | | (2.3-3) | (2.5-3.2) | (0.43-0.91) | |
| | Creatinine, mg/dL (IQR) | 0.94 (0.61-1.6) | 0.75 (0.62-1.18) | 1.34 (1.06-1.7) | ***0.016*** |
| | Blood urea nitrogen, mg/dL (IQR) | 27.9 (16.4-45.3) | 19 (12.3-29) | 1.01 (1-1.02) | ***0.01*** |
| | Hemoglobin, g/dL (IQR) | 9.3 (8.4-11.48) | 10.4 (8.9-11.9) | 0.92 (0.81-1.03) | 0.14 |
| | Sodium, mEq/L (IQR) | 141 (138-145) | 140 (138-143) | 1.03 (0.98-1.08) | 0.24 |
| | Potassium, mmol/L (IQR) | 3.7 (3.4-4) | 3.8 (3.5-4.2) | 0.52 (0.32-0.85) | ***0.007*** |
| | Phosphorus, mg/dl (IQR) | 3.14 (2.53-4.02) | 3.29 (2.48-4.11) | 0.98 (0.82-1.17) | 0.83 |
| | Magnesium, mg/dl (IQR) | 1.96 (1.72-2.19) | 1.99 (1.76-2.23) | 0.65 (0.32-1.32) | 0.21 |
| | Brain natriuretic peptide, pg/mL (IQR)** | 2089 (316-3823) | 1182 (425-3711) | 1 (0.99-1) | 0.82 |
| | pH | 7.42 (7.34-7.47) | 7.41 (7.36-7.47) | 0.7 (0.02-22.64) | 0.84 |
| | $pO_2$, mmHg (IQR) | 96 (80.5-124.5) | 106 (83-138) | 0.99 (0.99-1) | 0.29 |
| | $pCO_2$, mmHg (IQR) | 47 (41-54) | 45 (39-53) | 1 (0.99-1.03) | 0.4 |
| | Bicarbonate, mmol/L (IQR) | 30.6 (24.8-36.4) | 29 (24.9-34.8) | 1.02 (0.98-1.05) | 0.22 |
| | Lactate, mmol/L (IQR) | 1.2 (0.9-1.7) | 1.1 (0.8-1.7) | 0.91 (0.75-1.09) | 0.23 |
| | Ionized calcium, mmol/L (IQR) | 1.1 (1.03-1.16) | 1.09 (1.04-1.14) | 2.72 (0.14-53.7) | 0.51 |

| | | | | | |
|---|---|---|---|---|---|
| | Hyperglycemia (>180mg/dL) during 48h preceding extubation, n (%) | 52 (62.7%) | 146 (59.4%) | 1.15 (0.69-1.92) | 0.59 |
| **Vital signs (last value before extubation)** | | | | | |
| | Heart rate, bpm (IQR) | 91 (80-109) | 92 (81-107) | 1 (0.99-1.01) | 0.96 |
| | Systolic blood pressure, mmHg (IQR) | 142 (123-156) | 138 (121-158) | 1 (0.99-1.01) | 0.7 |
| | Diastolic blood pressure, mmHg (IQR) | 64 (56-74) | 66 (57-75) | 1 (0.98-1.01) | 0.64 |
| | Core Temperature, $^{o}$C (IQR) | 36.7 (36.2-37.4) | 36.9 (36.3-37.4) | 0.91 (0.67-1.25) | 0.57 |
| | Oxygen saturation, % (IQR) | 98 (96-100) | 98 (96-100) | 0.99 (0.88-1.1) | 0.8 |
| | Fraction of inspired oxygen, % (IQR) | 40 (35-40) | 40 (35-40) | 0.99 (0.95-1.05) | 0.94 |
| **Muscle related laboratory values (lowest value over the preceding 10 days)** | | | | | |
| | Alanine transaminase, IU/L (IQR) | 15 (9-21) | 20 (12-26) | 0.96 (0.93-0.98) | ***0.006*** |
| | Creatine phosphokinase, U/L (IQR)*** | 98 (38-257) | 142 (56-478) | 1 (0.99-1) | 0.46 |

Statistically significant values ($p< 0.05$) are italicized and bolded.

* APACHE-II score was recorded in 239 cases (72.6%)

** Brain natriuretic peptide values were available for 72 patients (21.9%)

*** Creatine phosphokinase values were available for 163 patients (49.5%)

OR- odds ratio; 95% CI- 95% confidence interval; IQR- interquartile range; COPD- chronic obstructive lung disease; APACHE-II score- Acute Physiology and Chronic Health Evaluation II
